# Supplementary material for: Alternative splicing in lung influences COVID-19 severity and respiratory diseases
Source: Nat Commun. 2023 Oct 4;14:6198. doi: 10.1038/s41467-023-41912-4 (PMC10550956; doi:10.1038/s41467-023-41912-4)
Supplement: Supplementary file 1 — Supplementary Information [file 41467_2023_41912_MOESM1_ESM.pdf]

**Alternative splicing in the lung influences COVID-19 severity and respiratory diseases.**

Nakanishi *et al.* 2023

# Supplementary Information

## Supplementary Figures

**Supplementary Fig. 1. Sashimi plots demonstrating the GTEx Leafcutter lung sQTL results.** ......... **2**

**Supplementary Fig. 2. LocusZoom plots demonstrating the colocalization of the genetic determinants of mRNA expression, RNA splicing levels in lung and COVID-19 outcomes.** ......... **5**

**Supplementary Fig. 1. Sashimi plots demonstrating the GTEx Leafcutter lung sQTL results.**

Sashimi plots to visualize the splice sites using the individual RNA-seq mapped bam files for lung samples (N=514) in GTEx v.8. Sashimi plots combine the information of read coverage along a gene with curves connecting splice sites supported by RNA-seq data. The mean number of reads supporting the splicing events per each genotype group are shown in the sashimi plots, which were adjusted for the average expression (counts per million: CPM) of the region including the cluster to which the index intronic junction belongs to and the exons at both ends. CPM was calculated by (the mapped read counts of the region / the total read counts) x $10^{-6}$ (**Supplementary Table 9**). The transcripts for all genes except for *OAS1* were annotated using gencode.v43.chr_patch_hapl_scaff.annotation.gtf (downloaded from https://www.gencodegenes.org/human/). For *OAS1*, we show only basic transcripts by using gencode.v43.basic.annotation.gtf to reduce the complexity of the figure. X-axis: chromosomal position (b38)

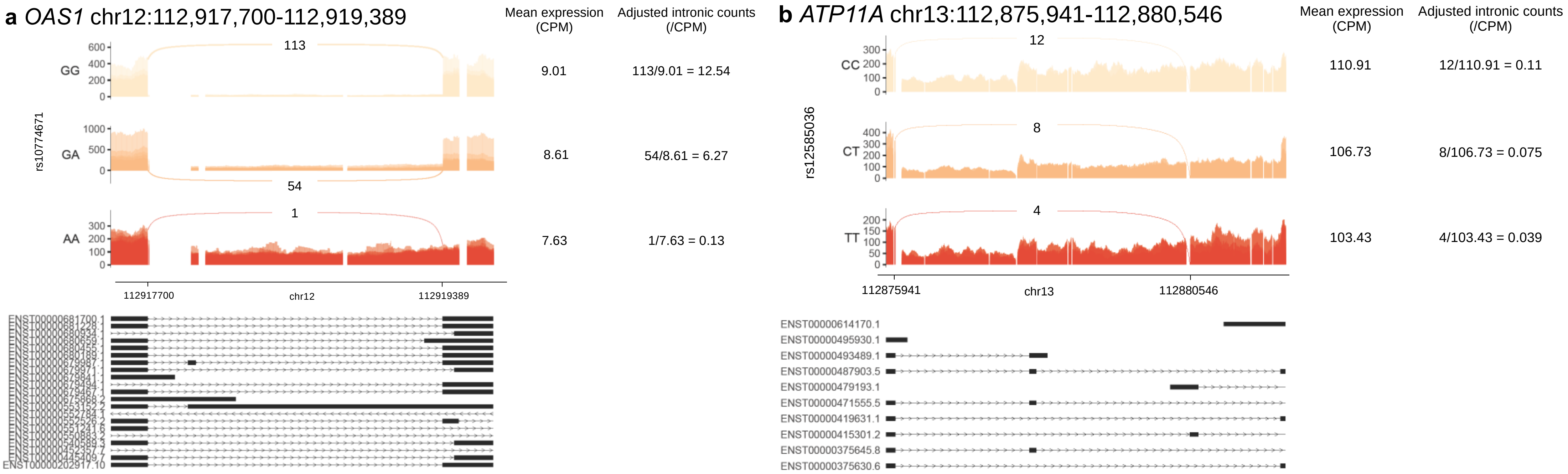


a) For the *OAS1* splicing event, 65 samples with a GG genotype, 255 samples with a GA genotype, and 194 samples with a AA genotype at rs10774671 were used to visualize the splicing event. These data show that the rs10774671-G allele is associated with increased excision of the intron junction at chr12:112,917,700-112,919,389.

b) For the *ATP11A* splicing event, 316 samples with a CC genotype, 177 samples with a CT genotype, and 21 samples with a TT genotype of rs12585036 were used to visualize the splicing event. These data show that the rs12585036-C allele is associated with increased excision of the intron junction at chr13:112,875,941-112,880,546.

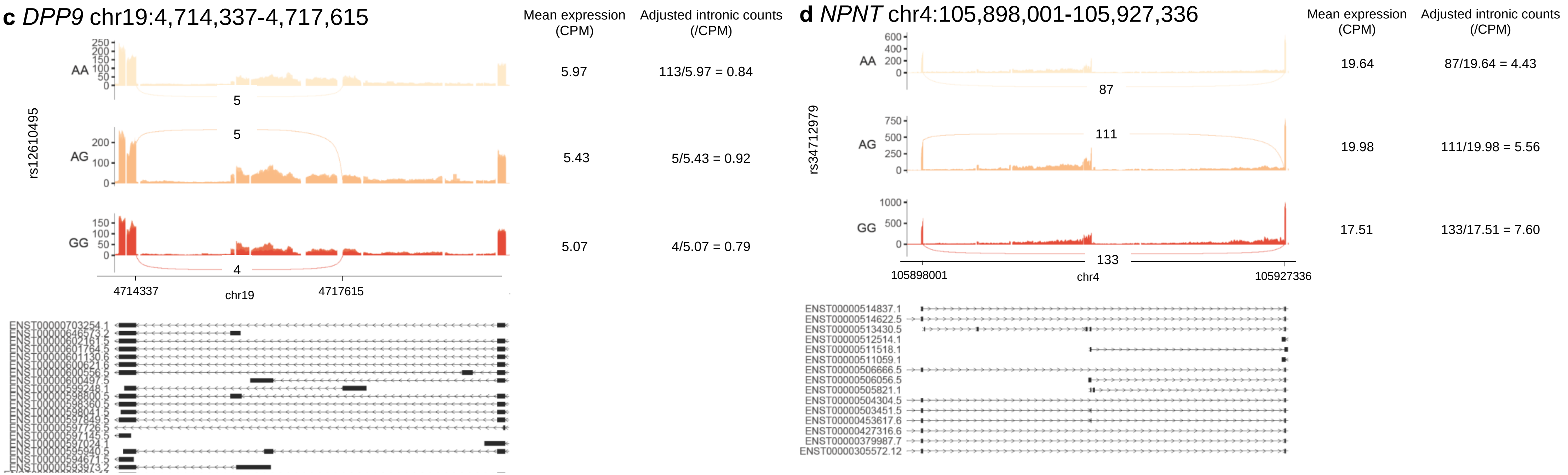


c) For the *DPP9* splicing event, 278 samples with a AA genotype, 191 samples with a AG genotype, and 45 samples with a GG genotype at rs12610495 were used to visualize the splicing event. These data show that the rs12610495-A allele was associated with increased excision of the intron junction at chr19:4,714,337-4,717,615.

d) For the *NPNT* splicing event, 33 samples with a AA genotype, 162 samples with a AG genotype, and 319 samples with a GG genotype at rs34712979 were used to visualize the splicing event. These data show that the rs34712979-G allele is associated with increased excision of the intron junction at chr4:105,898,001-105,927,336.

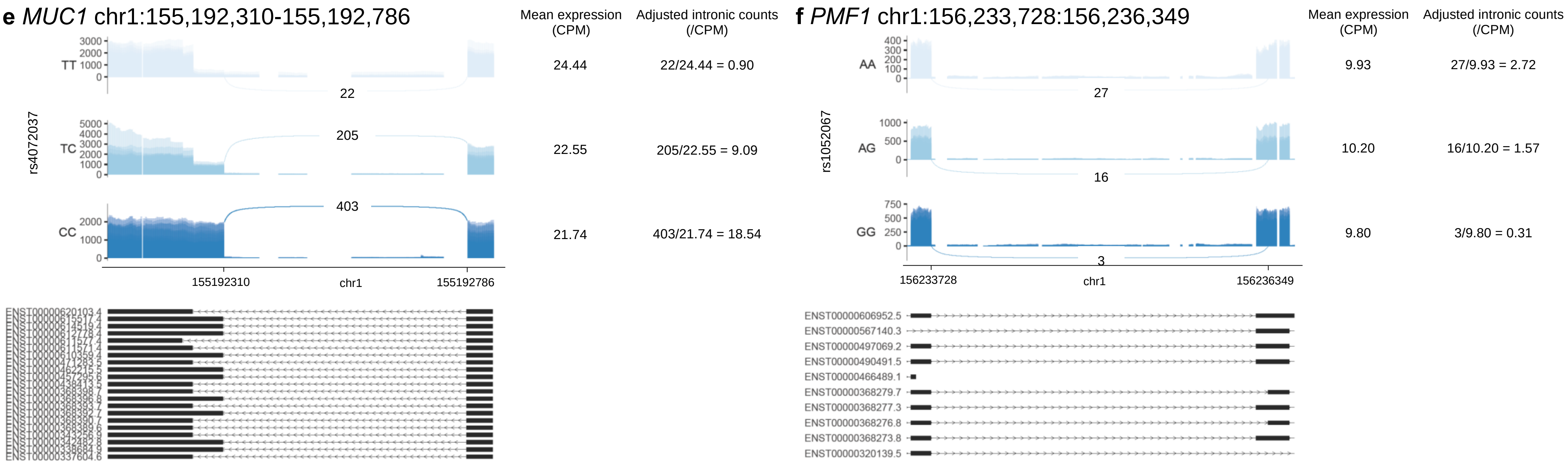


e) For the *MUC1* splicing event, 156 samples with a TT genotype, 251 samples with a TC genotype, and 107 samples with a CC genotype of rs4072037 were used to visualize the splicing event. These data show that the rs4072037-C allele is associated with increased excision of the intron junction at chr1:155,192,310-155,192,786.

f) For the *PMF1* splicing event, 37 samples with a AA genotype, 208 samples with a AG genotype, and 269 samples with a GG genotype of rs1052067 were used to visualize the splicing event. These data show that the rs1052067-A allele is associated with increased excision of the intron junction at chr1:156,233,728:156,236,349.

**Supplementary Fig. 2. LocusZoom plots demonstrating the colocalization of the genetic determinants of mRNA expression, RNA splicing levels in lung and COVID-19 outcomes.**

LocusZoom plots of e/sQTLs in lung, and COVID-19 outcomes within a 1MB region around each *cis*-sQTL in lung. Color shows LD ($r^2$) values in 1000G European ancestry to the *cis*-sQTL. a) *OAS1* b) *ATP11A* c) *DPP9* d) *NPNT* e) *MUC1* f) *PMF1.*

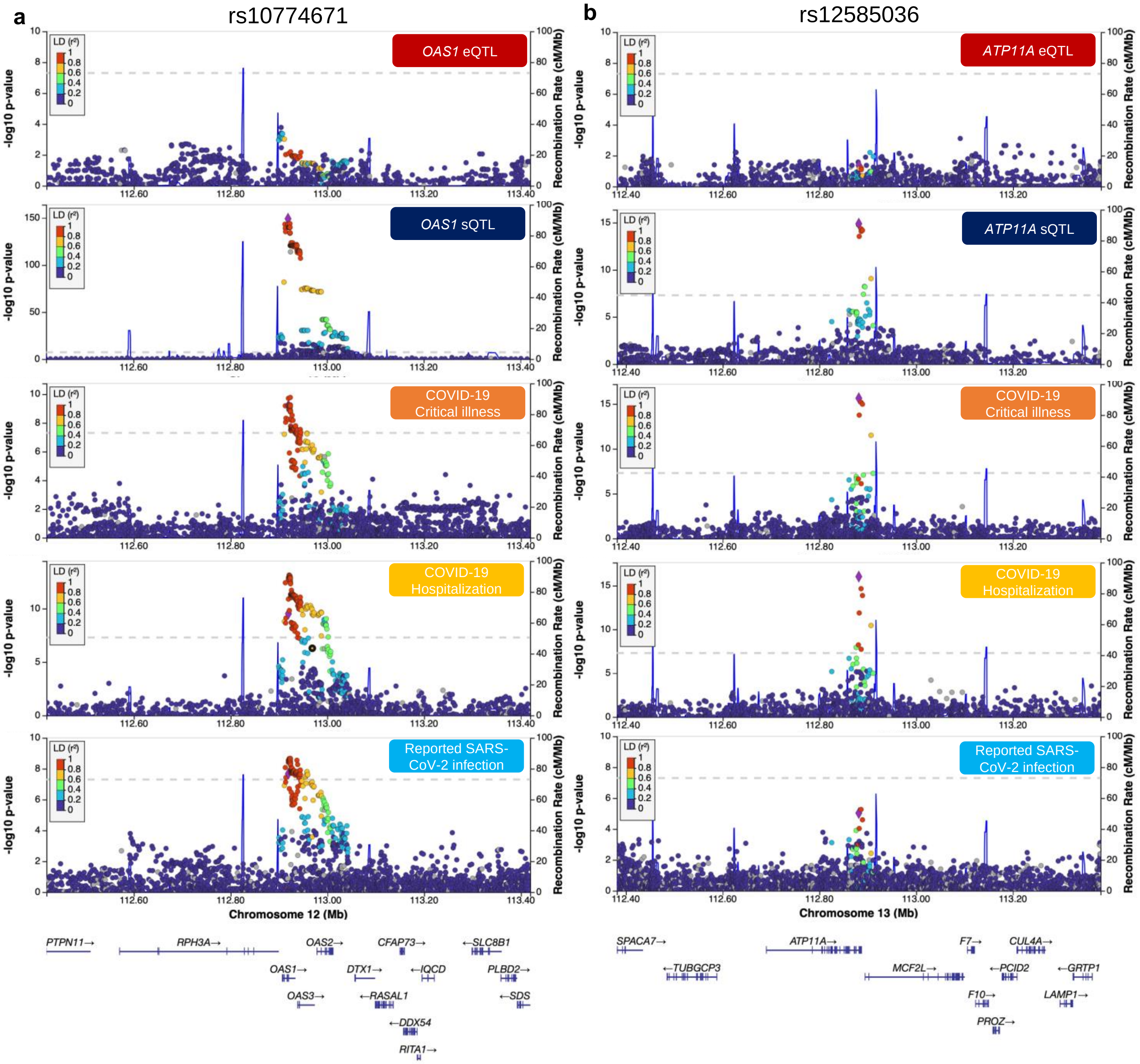

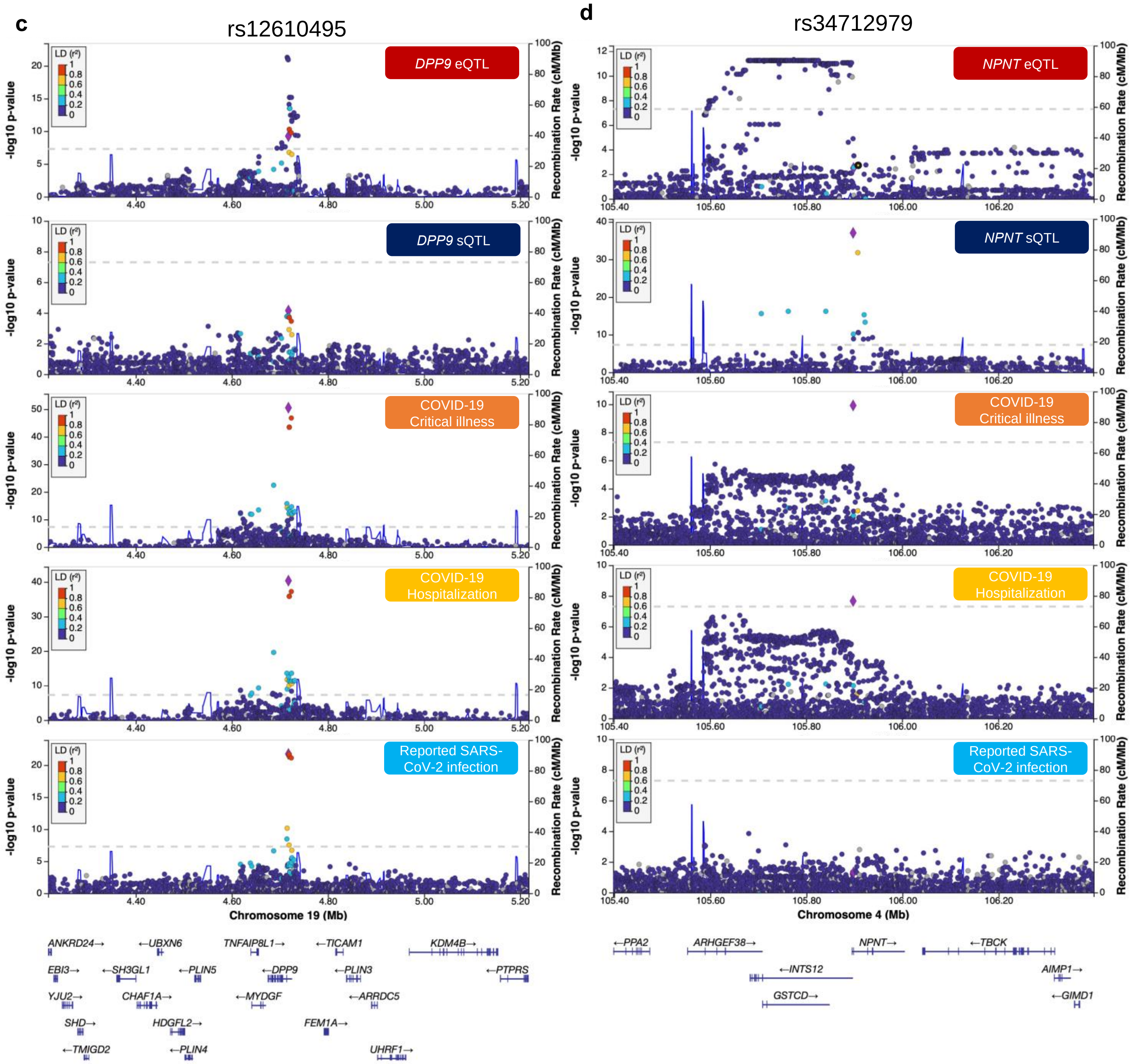
c
rs12610495
DPP9 eQTL
DPP9 sQTL
COVID-19 Critical illness
COVID-19 Hospitalization
Reported SARS-CoV-2 infection
-log10 p-value
Recombination Rate (cM/Mb)
LD (r²)
Chromosome 19 (Mb)
ANKRD24→
←UBXN6
TNFAIP8L1→
←TICAM1
KDM4B→
EBI3→
←SH3GL1
←PLIN5
←DPP9
←PLIN3
←PTPRS
YJU2→
CHAF1A→
←MYDGF
←ARRDC5
SHD→
HDGFL2→
FEM1A→
←TMIGD2
←PLIN4
UHRF1→
d
rs34712979
NPNT eQTL
NPNT sQTL
COVID-19 Critical illness
COVID-19 Hospitalization
Reported SARS-CoV-2 infection
Chromosome 4 (Mb)
←PPA2
ARHGEF38→
NPNT→
←TBCK
←INTS12
AIMP1→
GSTCD→
←GIMD1

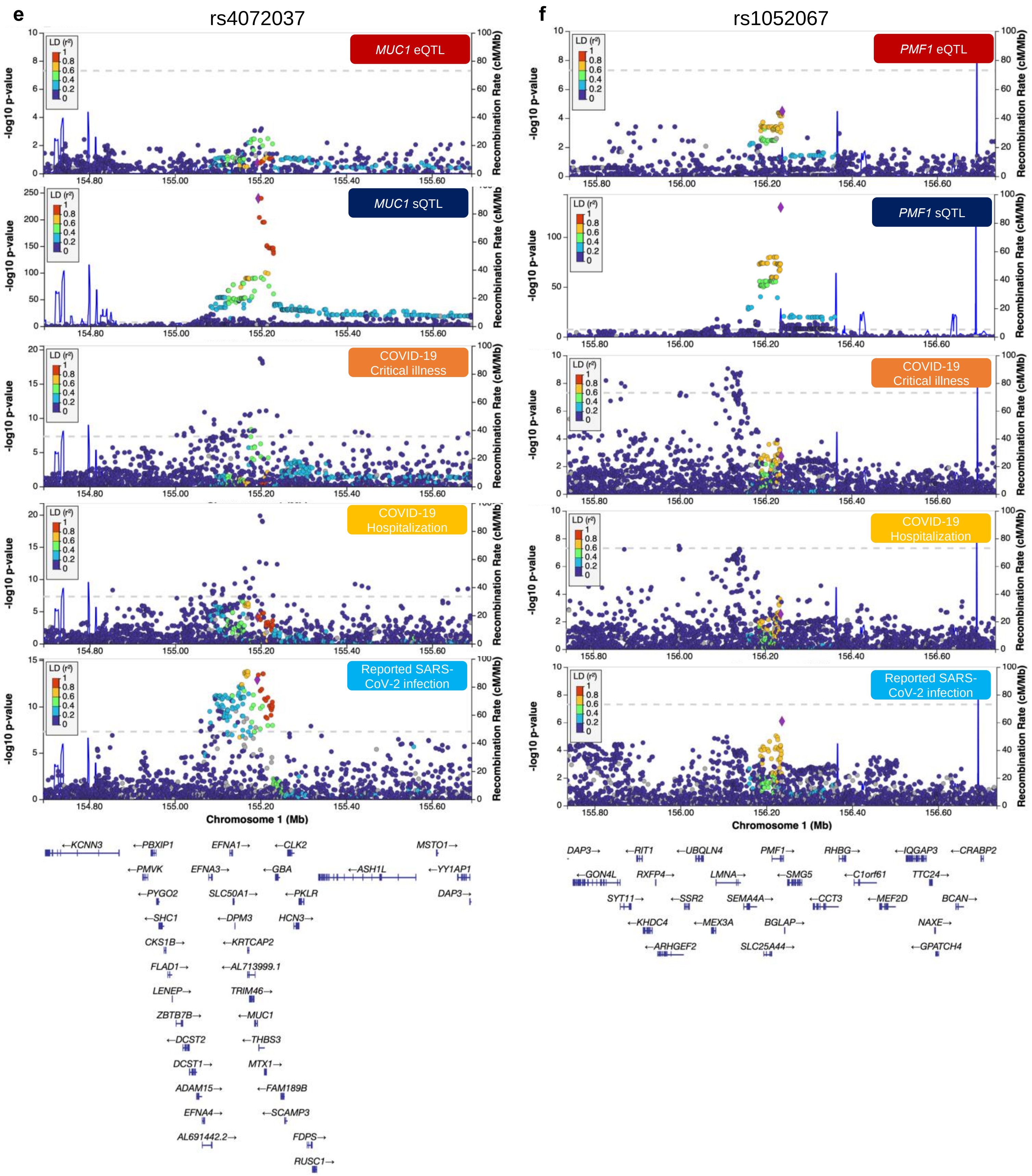

e
rs4072037
MUC1 eQTL
MUC1 sQTL
COVID-19 Critical illness
COVID-19 Hospitalization
Reported SARS-CoV-2 infection
-log10 p-value
Recombination Rate (cM/Mb)
Chromosome 1 (Mb)
f
rs1052067
PMF1 eQTL
PMF1 sQTL
COVID-19 Critical illness
COVID-19 Hospitalization
Reported SARS-CoV-2 infection
-log10 p-value
Recombination Rate (cM/Mb)
Chromosome 1 (Mb)
