# Supplementary material for: Alternative splicing in lung influences COVID-19 severity and respiratory diseases
Source: Nat Commun. 2023 Oct 4;14:6198. doi: 10.1038/s41467-023-41912-4 (PMC10550956; doi:10.1038/s41467-023-41912-4)
Supplement: Supplementary file 3 — Description of Additional Supplementary Files [file 41467_2023_41912_MOESM3_ESM.pdf]

Description of Additional Supplementary Files

File Name: Supplementary Data 1
Description: Two sample Mendelian randomization results of alternative splicing in lung using independent cis-sQTLs from GTEx consortium.
exposure: “chrX:POS1:POS2:clusterID:ensembleID” represents the excised intron region, the clusters each intron belongs to (defined in GTEx), and the gene ensemble ID.
hgnc_symbol: human gene nomenclature, Method: Wald ratio if single variants, and Inverse variance weighted if multiple variants.
Variant list: the list of instrumental variables (chromosome:position[b38]:non effect allele:effect allele).
OR: Odds ratio per standard deviation of intron excision ratios as quantified by LeafCutter, LL: Lower limit of 95%CI, UL: upper limit of 95%CI
p-value: associated with the estimate using a normal distribution (Two-sided).
Significant MR findings (p-value<0.05/27230) for outcome "Critical illness"
Significant MR findings (p-value<0.05/27230) for outcome "Hospitalization"
Significant MR findings (p-value<0.05/27230) for outcome "Reported infection"

File Name: Supplementary Data 2
Description: Two sample Mendelian randomization results of alternative splicing in whole blood using independent cis-sQTLs from GTEx consortium.
exposure: “chrX:POS1:POS2:clusterID:ensembleID” represents the excised intron region, the clusters each intron belongs to (defined in GTEx), and the gene ensemble ID.
hgnc_symbol: human gene nomenclature, Method: Wald ratio if single variants, and Inverse variance weighted if multiple variants.
Variant list: the list of instrumental variables (chromosome:position[b38]:non effect allele:effect allele).
OR: Odds ratio per standard deviation of intron excision ratios as quantified by LeafCutter, LL: Lower limit of 95%CI, UL: upper limit of 95%CI
p-value: associated with the estimate using a normal distribution (Two-sided).
Significant MR findings (p-value<0.05/27230) for outcome "Critical illness"
Significant MR findings (p-value<0.05/27230) for outcome "Hospitalization"
Significant MR findings (p-value<0.05/27230) for outcome "Reported infection"

File Name: Supplementary Data 3
Description: Two sample Mendelian randomization results for hospitalization phenotype compared within COVID-19 cases
(“hospitalization vs non-hospitalization amongst individuals with laboratory-confirmed SARS-CoV-2 infection”, which corresponds to B1 phenotype in COVID-19 HGI.
exposure: “chrX:POS1:POS2:clusterID:ensembleID” represents the excised intron region, the clusters each intron belongs to (defined in GTEx), and the gene ensemble ID.
hgnc_symbol: human gene nomenclature, Method: Wald ratio if single variants, and Inverse variance weighted if multiple variants.
Variant list: the list of instrumental variables (chromosome:position[b38]:non effect allele:effect allele).

OR: Odds ratio per standard deviation of intron excision ratios as quantified by LeafCutter, LL: Lower limit of 95%CI, UL: upper limit of 95%CI
Significant MR findings (p-value<0.05)

File Name: Supplementary Data 4
Description: Colocalization analyses to test if GWAS for transcriptional splicing in lung and COVID-19 outcomes share genetic signals.
exposure: “chrX:POS1:POS2:clusterID:ensembleID” represents the excised intron region, the clusters each intron belongs to (defined in GTEx), and the gene ensemble ID.
NSNP: Number of SNPs used for colocalization, PP.H{0..4}.abf: posterior probability (PP) of the shared causal variant hypothesis H{0..4} in the colocalization analyses.
Hypothesis 0 (H0):no association with either trait in the region, H1: association with trait 1 only, H2: association with trait 2 only,
H3: both traits are associated, but have different single causal variants, H4: both traits are associated and share the same single causal variant
Significant MR findings (p-value<0.05/27230) and high colocalization posterior probability (PP.H4.abf > 0.8) for outcome "Critical illness"
Significant MR findings (p-value<0.05/27230) and high colocalization posterior probability (PP.H4.abf > 0.8) for outcome "Hospitalization"
Significant MR findings (p-value<0.05/27230) and high colocalization posterior probability (PP.H4.abf > 0.8) for outcome "Reported infection"

File Name: Supplementary Data 5
Description: Sensitivity analyses for alternative splicing in whole blood using individuals of European ancestry.
exposure: “chrX:POS1:POS2:clusterID:ensembleID” represents the excised intron region, the clusters each intron belongs to (defined in GTEx), and the gene ensemble ID.
NSNP: Number of SNPs used for colocalization, PP.H{0..4}.abf: posterior probability (PP) of the shared causal variant hypothesis H{0..4} in the colocalization analyses.
Hypothesis 0 (H0):no association with either trait in the region, H1: association with trait 1 only, H2: association with trait 2 only,
H3: both traits are associated, but have different single causal variants, H4: both traits are associated and share the same single causal variant
Significant MR findings (p-value<0.05/27230) and high colocalization posterior probability (PP.H4.abf > 0.8) for outcome "Critical illness"
Significant MR findings (p-value<0.05/27230) and high colocalization posterior probability (PP.H4.abf > 0.8) for outcome "Hospitalization"
Significant MR findings (p-value<0.05/27230) and high colocalization posterior probability (PP.H4.abf > 0.8) for outcome "Reported infection"

File Name: Supplementary Data 6
Description: Pleiotropic eQTL effects of the sQTLs used as instruments for the significant MR findings with high colocalization.
EA: Effect Allele, NEA: Non-effect allele

File Name: Supplementary Data 7
Description: MR and colocalization analysis for gene expression in lung and COVID-19 outcomes.
hgnc_symbol: human gene nomenclature, Method: Wald ratio if single variants, and Inverse variance weighted if multiple variants.
Variant list: the list of instrumental variables (chromosome:position[b38]:non effect allele:effect allele).
OR: Odds ratio per standard deviation of intron excision ratios as quantified by LeafCutter, LL: Lower limit of 95%CI, UL: upper limit of 95%CI
p-value: associated with the estimate using a normal distribution (Two-sided).
NSNP: Number of SNPs used for colocalization, PP.H{0..4}.abf: posterior probability (PP) of the shared causal variant hypothesis H{0..4} in the colocalization analyses.
Hypothesis 0 (H0):no association with either trait in the region, H1: association with trait 1 only, H2: association with trait 2 only,
H3: both traits are associated, but have different single causal variants, H4: both traits are associated and share the same single causal variant
High colocalization (PP.H4.abf > 0.8) for outcome "Critical illness"
High colocalization (PP.H4.abf > 0.8) for outcome "Hospitalization"
High colocalization (PP.H4.abf > 0.8) for outcome "Reported infection"

File Name: Supplementary Data 8
Description: MR and colocalization analysis for gene expression in whole blood and COVID-19 outcomes.
hgnc_symbol: human gene nomenclature, Method: Wald ratio if single variants, and Inverse variance weighted if multiple variants.
Variant list: the list of instrumental variables (chromosome:position[b38]:non effect allele:effect allele).
OR: Odds ratio per standard deviation of intron excision ratios as quantified by LeafCutter, LL: Lower limit of 95%CI, UL: upper limit of 95%CI
p-value: associated with the estimate using a normal distribution (Two-sided).
NSNP: Number of SNPs used for colocalization, PP.H{0..4}.abf: posterior probability (PP) of the shared causal variant hypothesis H{0..4} in the colocalization analyses.
Hypothesis 0 (H0):no association with either trait in the region, H1: association with trait 1 only, H2: association with trait 2 only,
H3: both traits are associated, but have different single causal variants, H4: both traits are associated and share the same single causal variant
High colocalization (PP.H4.abf > 0.8) for outcome "Critical illness"
High colocalization (PP.H4.abf > 0.8) for outcome "Hospitalization"
High colocalization (PP.H4.abf > 0.8) for outcome "Reported infection"

File Name: Supplementary Data 9
Description: Genomic ranges used to calculate CPM.
