# Supplementary material for: Alternative splicing in lung influences COVID-19 severity and respiratory diseases
Source: Nat Commun. 2023 Oct 4;14:6198. doi: 10.1038/s41467-023-41912-4 (PMC10550956; doi:10.1038/s41467-023-41912-4)
Supplement: Supplementary file 5 — Reporting Summary [file 41467_2023_41912_MOESM5_ESM.pdf]

nature portfolio

Corresponding author(s): Tomoko Nakanishi, J. Brent Richards

Last updated by author(s): Aug 18, 2023

Reporting Summary

Nature Portfolio wishes to improve the reproducibility of the work that we publish. This form provides structure for consistency and transparency in reporting. For further information on Nature Portfolio policies, see our Editorial Policies and the Editorial Policy Checklist.

Statistics

For all statistical analyses, confirm that the following items are present in the figure legend, table legend, main text, or Methods section.

n/a	Confirmed	
☐	☒	The exact sample size (*n*) for each experimental group/condition, given as a discrete number and unit of measurement
☐	☒	A statement on whether measurements were taken from distinct samples or whether the same sample was measured repeatedly
☐	☒	The statistical test(s) used AND whether they are one- or two-sided<br>*Only common tests should be described solely by name; describe more complex techniques in the Methods section.*
☒	☐	A description of all covariates tested
☐	☒	A description of any assumptions or corrections, such as tests of normality and adjustment for multiple comparisons
☐	☒	A full description of the statistical parameters including central tendency (e.g. means) or other basic estimates (e.g. regression coefficient) AND variation (e.g. standard deviation) or associated estimates of uncertainty (e.g. confidence intervals)
☐	☒	For null hypothesis testing, the test statistic (e.g. *F*, *t*, *r*) with confidence intervals, effect sizes, degrees of freedom and *P* value noted<br>*Give P values as exact values whenever suitable.*
☐	☒	For Bayesian analysis, information on the choice of priors and Markov chain Monte Carlo settings
☒	☐	For hierarchical and complex designs, identification of the appropriate level for tests and full reporting of outcomes
☐	☒	Estimates of effect sizes (e.g. Cohen's *d*, Pearson's *r*), indicating how they were calculated

Our web collection on statistics for biologists contains articles on many of the points above.

Software and code

Policy information about availability of computer code

Data collection	No software was used in data collection.
Data analysis	All code for data management and analysis is archived online at github.com/richardslab/COVID19-sQTLMR for review and reuse. We used ggsashimi (https://github.com/guigolab/ggsashimi) R package v1.1.5 to visualize splice junction usage per sQTL genotype. We used TwoSampleMR v0.5.6 R package68 to run two-sample mendelian randomization (MR) analyses. Colocalization analyses were performed using coloc v5.1.0.1 R package. We visualized RNA single cell type tissue cluster data using corrplot v0.92 R package.

For manuscripts utilizing custom algorithms or software that are central to the research but not yet described in published literature, software must be made available to editors and reviewers. We strongly encourage code deposition in a community repository (e.g. GitHub). See the Nature Portfolio guidelines for submitting code & software for further information.

Data

Policy information about availability of data

All manuscripts must include a data availability statement. This statement should provide the following information, where applicable:

- Accession codes, unique identifiers, or web links for publicly available datasets
- A description of any restrictions on data availability
- For clinical datasets or third party data, please ensure that the statement adheres to our policy

Summary statistics for eQTLs and sQTLs from GTEx v823 are available in the GTEx website (gtexportal.org/home/datasets). The GTEx protected access data are

available under restricted access, access can be obtained through dbGaP (application ID: 32756). Summary statistics for the COVID-19 outcomes release 7 are available in the COVID-19 Host Genetics Initiative website72 (https://www.covid19hg.org/results/r7/). The consensus transcript expression levels and RNA single cell type tissue cluster data are available at the Human Protein Atlas website (www.proteinatlas.org/about/download). RNA single cell type tissue cluster data for lung and PBMC was originally obtained from GSE13014870 and GSE11284571, respectively. The processed single-cell RNA expression profile of 23 lung COVID-19 autopsy donor tissue sample are available at the Gene Expression Omnibus (GEO, https://www.ncbi.nlm.nih.gov/geo/) under accession code GSE17166821.

Human research participants

Policy information about studies involving human research participants and Sex and Gender in Research.

Reporting on sex and gender	All GWAS summaries were adjusted for sex in their original papers. We could not perform sex-stratified analyses due to the lack of the availability of the sex-stratified GWAS summaries.
Population characteristics	The e/sQTL GWAS summary obtained from the GTEx consortium are based on N=452 of European American ancestry for lung and N=570 of European American ancestry for whole blood. The COVID-19 GWAS summary obtained from the COVID-19 Host Genetics Initiative consist of 122,616 cases and 2,475,240 controls of European ancestry.
Recruitment	For the GTEx protected access data, we have used the samples whose whole exome sequence and RNA sequence data are both available. Not applicable for the use of other publicly available data.
Ethics oversight	We have applied dbGaP to access the GTEx protected access data with application ID 32756 (IRB board at Jewish General Hospital: 2024-3794)

Note that full information on the approval of the study protocol must also be provided in the manuscript.

Field-specific reporting

Please select the one below that is the best fit for your research. If you are not sure, read the appropriate sections before making your selection.

☒ Life sciences ☐ Behavioural & social sciences ☐ Ecological, evolutionary & environmental sciences

For a reference copy of the document with all sections, see nature.com/documents/nr-reporting-summary-flat.pdf

Life sciences study design

All studies must disclose on these points even when the disclosure is negative.

Sample size	We did not use any statistical methods to predetermine sample size. We have chosen to use the GWAS summaries of the largest sample sizes available for each outcomes.
Data exclusions	In MR analyses, we selected cis-sQTLs as instrumental variables, as cis- genetic variants that reside close to the genes are more likely to have an effect on the outcomes by directly influencing the alternative splicing, thus reducing potential horizontal pleiotropy. Palindromic cis-sQTLs with minor allele frequencies (MAF) >0.42 were removed prior to MR to prevent allele-mismatches. We also removed genetic variants within MHC region to reduce the risk of bias from LD.
Replication	We did not attempt to replicate our results by constructing another dataset, given that the COVID-19 HGI GWASs are the largest meta-analyses for COVID-19 outcomes and no other external dataset with the equivalent sample size exists.This
Randomization	We did not perform any randomizaton, as this is not a clinical trial study.
Blinding	We did not perform any blinding, as this is not a clinical trial study.

Reporting for specific materials, systems and methods

We require information from authors about some types of materials, experimental systems and methods used in many studies. Here, indicate whether each material, system or method listed is relevant to your study. If you are not sure if a list item applies to your research, read the appropriate section before selecting a response.

Materials & experimental systems

n/a	Involved in the study
☒	☐ Antibodies
☒	☐ Eukaryotic cell lines
☒	☐ Palaeontology and archaeology
☒	☐ Animals and other organisms
☒	☐ Clinical data
☒	☐ Dual use research of concern

Methods

n/a	Involved in the study
☒	☐ ChIP-seq
☒	☐ Flow cytometry
☒	☐ MRI-based neuroimaging
